# Supplementary material for: Creation of chiral interface channels for quantized transport in magnetic topological insulator multilayer heterostructures
Source: Nat Commun. 2023 Feb 11;14:770. doi: 10.1038/s41467-023-36488-y (PMC9918724; doi:10.1038/s41467-023-36488-y)
Supplement: Supplementary file 2 — Supplementary Information [file 41467_2023_36488_MOESM2_ESM.pdf]

Supplementary Information

Creation of Chiral Interface Channels for Quantized Transport in Magnetic Topological Insulator Multilayer Heterostructures

Yi-Fan Zhao[1,5], Ruoxi Zhang[1,5], Jiaqi Cai[2], Deyi Zhuo[1], Ling-Jie Zhou[1], Zi-Jie Yan[1], Moses H. W. Chan[1], Xiaodong Xu[2,3], and Cui-Zu Chang[1,4]

[1] Department of Physics, The Pennsylvania State University, University Park, PA 16802, USA

[2] Department of Physics, University of Washington, Seattle, WA 98195, USA

[3] Department of Material Science and Engineering, University of Washington, Seattle, WA 98195, USA

[4] Materials Research Institute, The Pennsylvania State University, University Park, PA 16802, USA

[5] These authors contributed equally: Yi-Fan Zhao and Ruoxi Zhang

Corresponding authors: cxc955@psu.edu (C.-Z. C.).

Content:

I. Supplementary Figures

II. Supplementary Notes

1. **Quantized transport in the junction between *C*=1 and *C*=-1 QAH insulators**
2. **Nonuniform thickness influence on CICs**
3. **Quantized transport behavior in electron-doped regime**
4. **Calculating the quantized transport in QAH junctions based on the Landauer–Büttiker formalism**

Supplementary References

I. Supplementary Figures

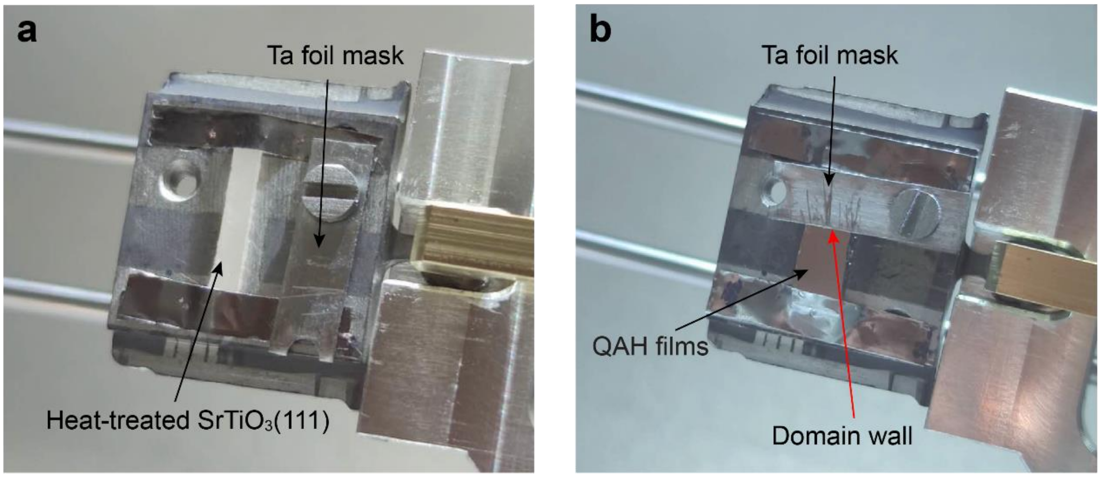

Supplementary Fig. 1| The *in-situ* mechanical mask used in our experiments. a, A custom-designed flag-style sample holder with a Ta foil mask. A 2 mm ×10 mm heat-treat $SrTiO_3$(111) is mounted in this sample holder. **b,** The QAH insulator junction is synthesized in magnetic TI/TI multilayer heterostructures by employing the *in-situ* Ta foil mask to cover half of the sample.

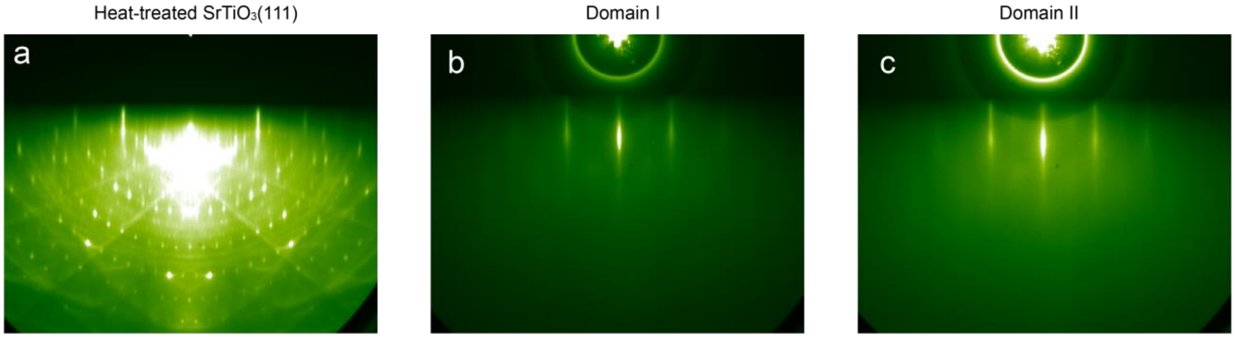

Supplementary Fig. 2| RHEED patterns of the junction between *C* = 1 and *C* = -1 QAH insulators. a, Heated-treated $SrTiO_3(111)$ substrate. **b,** Domain I: 2 QL $(Bi, Sb)_{1.74}Cr_{0.26}Te_3$/2 QL $(Bi, Sb)_2Te_3$/2 QL $(Bi, Sb)_{1.74}Cr_{0.26}Te_3$, **c,** Domain II: 2 QL $(Bi, Sb)_{1.78}V_{0.22}Te_3$/2 QL $(Bi, Sb)_{1.74}Cr_{0.26}Te_3$/2 QL $(Bi, Sb)_2Te_3$/2 QL $(Bi, Sb)_{1.74}Cr_{0.26}Te_3$.

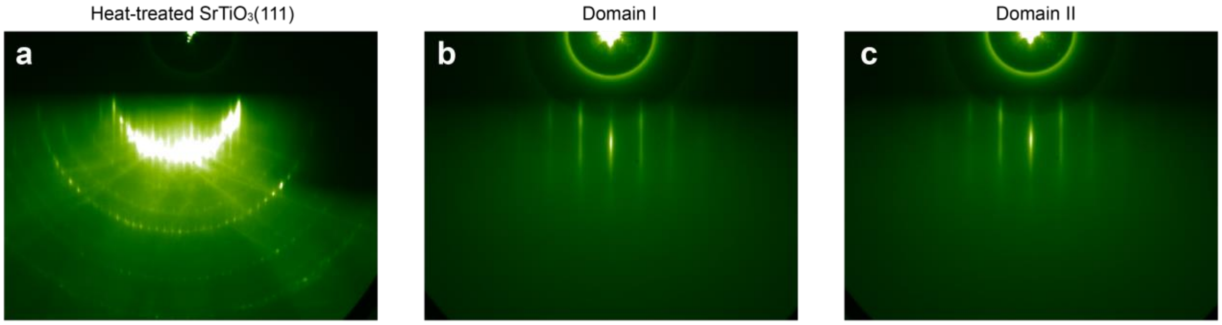

Supplementary Fig. 3| RHEED patterns of the junctions between *C* = 1 and *C* = 2 QAH insulators a, Heated-treated $SrTiO_3(111)$ substrate. **b,** Domain I: 3 QL $(Bi, Sb)_{1.74}Cr_{0.26}Te_3$/4 QL $(Bi, Sb)_2Te_3$/3 QL $(Bi, Sb)_{1.74}Cr_{0.26}Te_3$. **c,** Domain II: [3 QL $(Bi, Sb)_{1.74}Cr_{0.26}Te_3$/4 QL $(Bi, Sb)_2Te_3]_2$/3 QL $(Bi, Sb)_{1.74}Cr_{0.26}Te_3$.

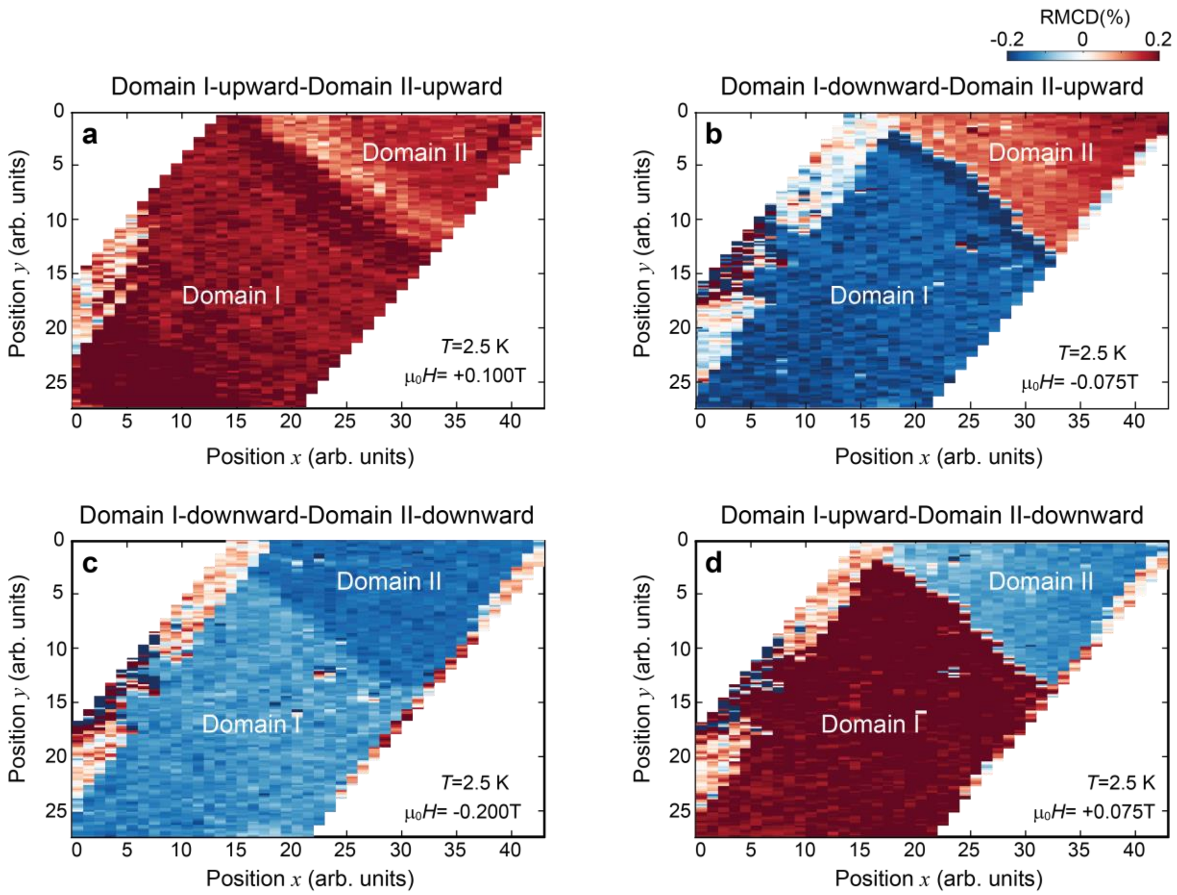

Supplementary Fig. 4| RMCD maps of the junction between *C* = 1 to *C* = -1 QAH insulators under different magnetic DW configurations. a, Domain I-upward-Domain II-upward state at $\mu_0 H_c \sim +0.100$ T. **b,** Domain I-downward-Domain II-upward state at $\mu_0 H_c \sim -0.075$ T. **c,** Domain I-downward-Domain II-downward state at $\mu_0 H_c \sim -0.200$ T. **d,** Domain I-upward-Domain II-downward state at $\mu_0 H_c \sim +0.075$ T. These RMCD maps are obtained under the following scanning direction of the magnetic field: +0.500 T to +0.100 T (a), +0.100 T to -0.075 T (b), -0.075 T to -0.200 T (c), and -0.200T to +0.075T (d).

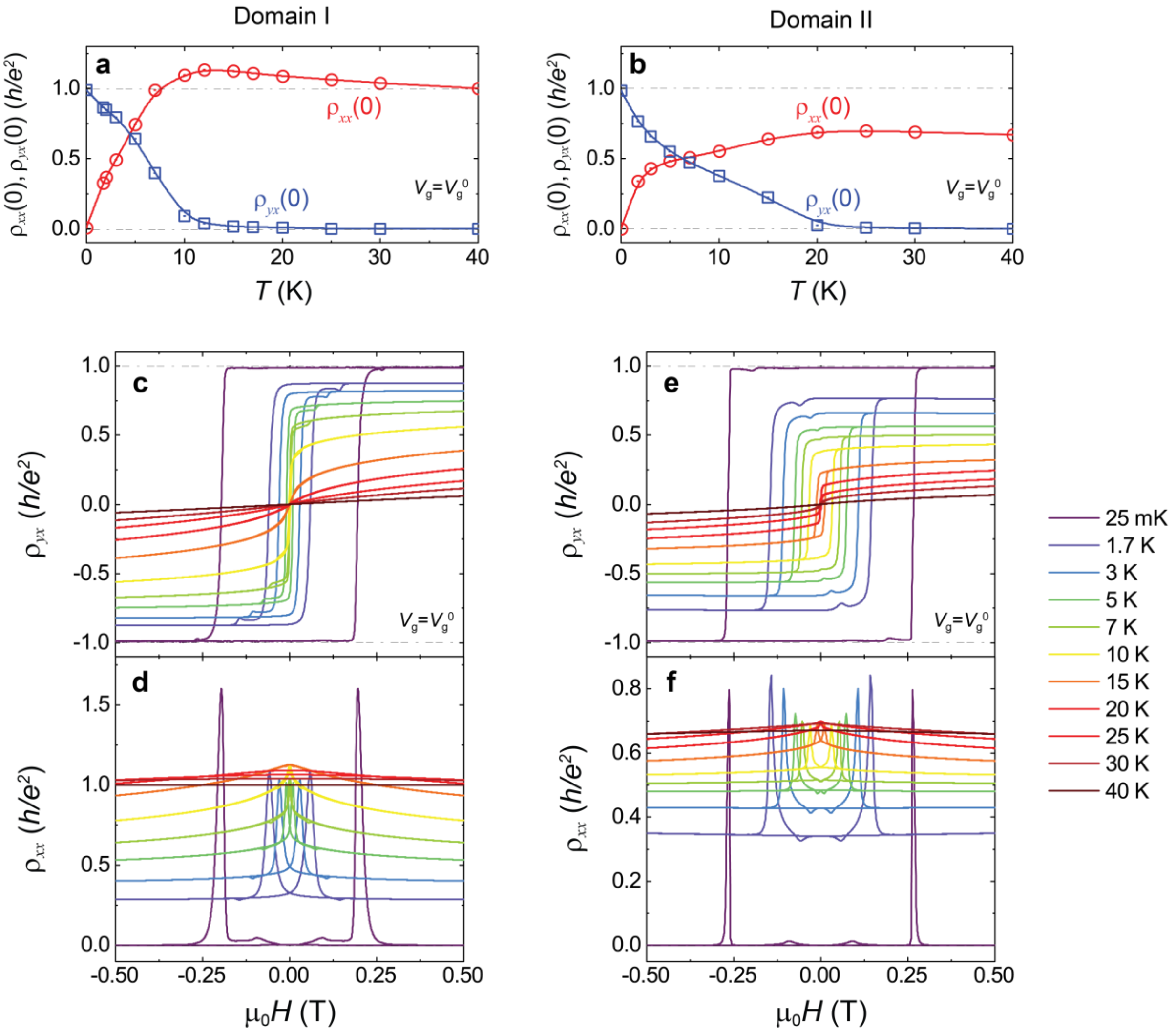

Supplementary Fig. 5| More transport results of Domain I and Domain II in the junction between *C* =1 QAH and *C* = -1 QAH insulators. a, b, Temperature dependence of ρ_{yx} (0) (blue squares) and ρ_{xx} (0) (red circles) of Domain I and Domain II. All measurements are performed at $\mu_0H = 0$ T after magnetic training. **c**, **d**, μ_0H dependence of ρ_{yx} (**c**) and ρ_{xx} (**d**) of Domain I measured at different temperatures and $V_g = V_g^0$. The Curie temperature of Domain I T_{C1} is ~18 K. **e**, **f**, Same as in (**c**) and (**d**), but for Domain II. The Curie temperature of Domain II T_{C2} is ~27 K.

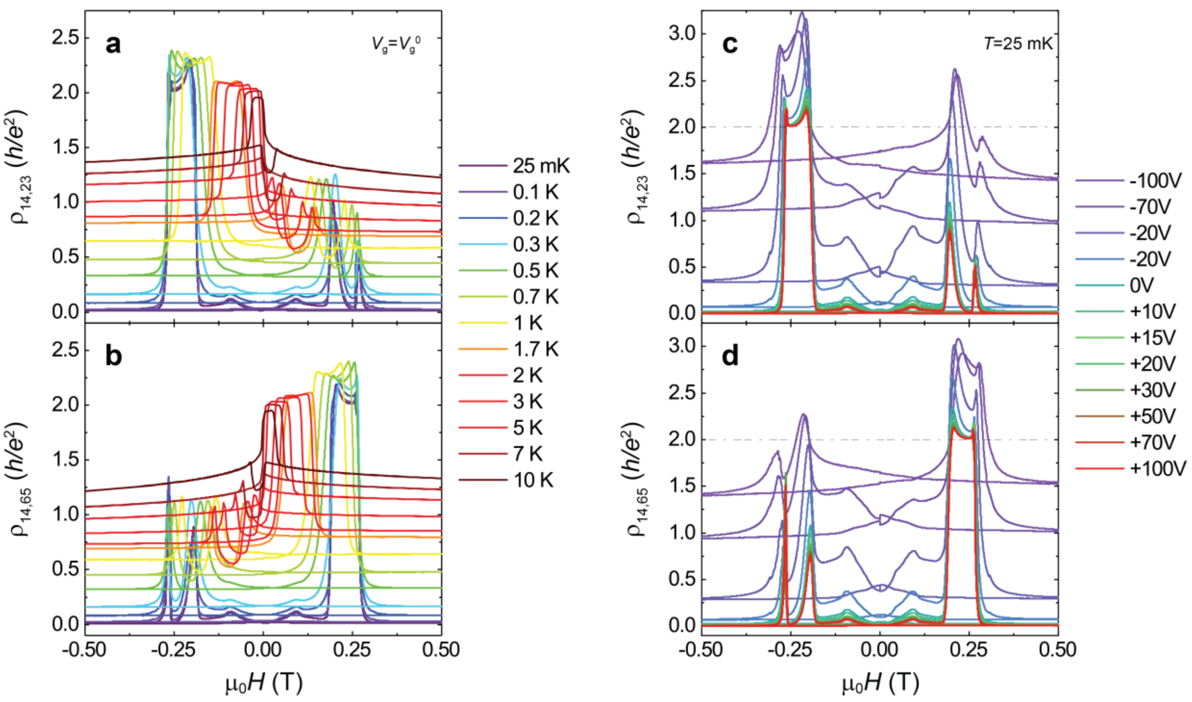

Supplementary Fig. 6| More transport results along the junction between C =1 QAH and C = -1 QAH insulators. a,**b**, $\mu_0 H$ dependence of $\rho_{14,23}$ (**a**)and $\rho_{14,65}$ (**b**) measured at different temperatures and $V_g = V_g^0$. **c**, **d**, $\mu_0 H$ dependence of $\rho_{14,23}$ (**c**) and $\rho_{14,65}$ (**d**) measured at different gate voltages V_gs and T = 25 mK. The charge neutral point V_g^0 ~ +15 V. Schematic of the device is shown in Fig. 2a of the main text.

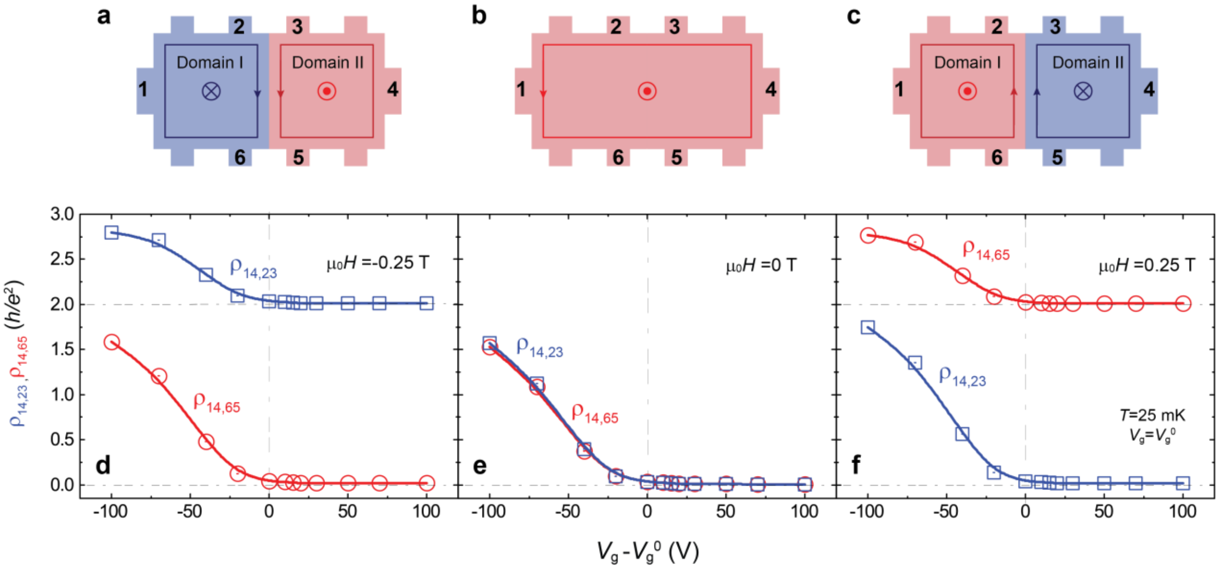

Supplementary Fig. 7| Demonstration of CICs with quantized transport along the junction between *C* =1 QAH and *C* = -1 QAH insulators. a-c, Schematics of chiral edge/interface channels under different DW configurations, which are created by tuning the external $\mu_0 H$. **d-f,** Gate (V_g-V_g^0) dependence of $\rho_{14,23}$ (blue) and $\rho_{14,65}$ (red) with the DW configurations in (**a** to **c**). All measurements are performed at T =25 mK.

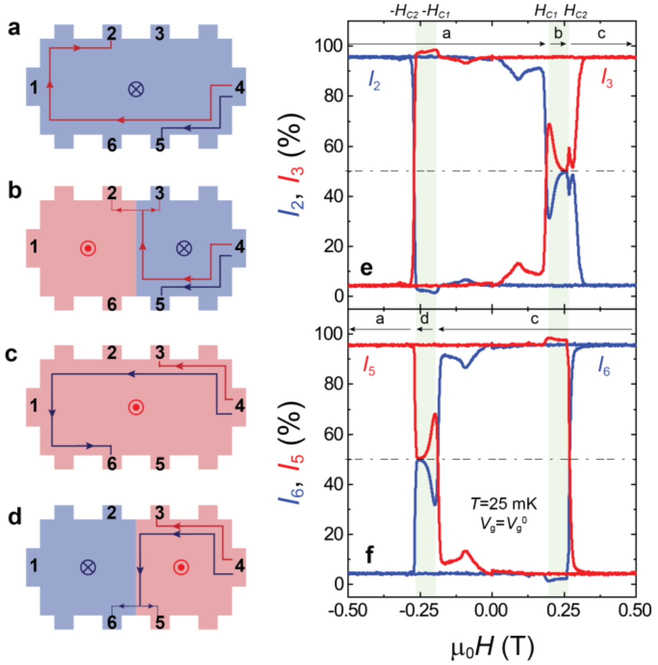

Supplementary Fig. 8| Chiral edge current distributor of the junction between C = 1 QAH and C = -1 QAH insulators when a current is injected from contact 4. a-d, Schematics of chiral edge/interface current under different DW configurations, which are created by tuning external $\mu_0 H$. When a current of ~1 nA is injected from contact 4, the drain current measured at contact 2 or 3 with other floating contacts is shown in red, while the drain current measured at contact 5 or 6 with other floating contacts is shown in blue. **e,** $\mu_0 H$ dependence of normalized drain current for contact 2 (blue) and 3 (red). **f,** $\mu_0 H$ dependence of normalized drain current for contact 5 (red) and 6 (blue). All measurements are performed at $V_g = V_g^0$ and T = 25 mK. The arrows in (e and f) indicate the magnetic field sweep directions and label the DW configurations in (a to d).

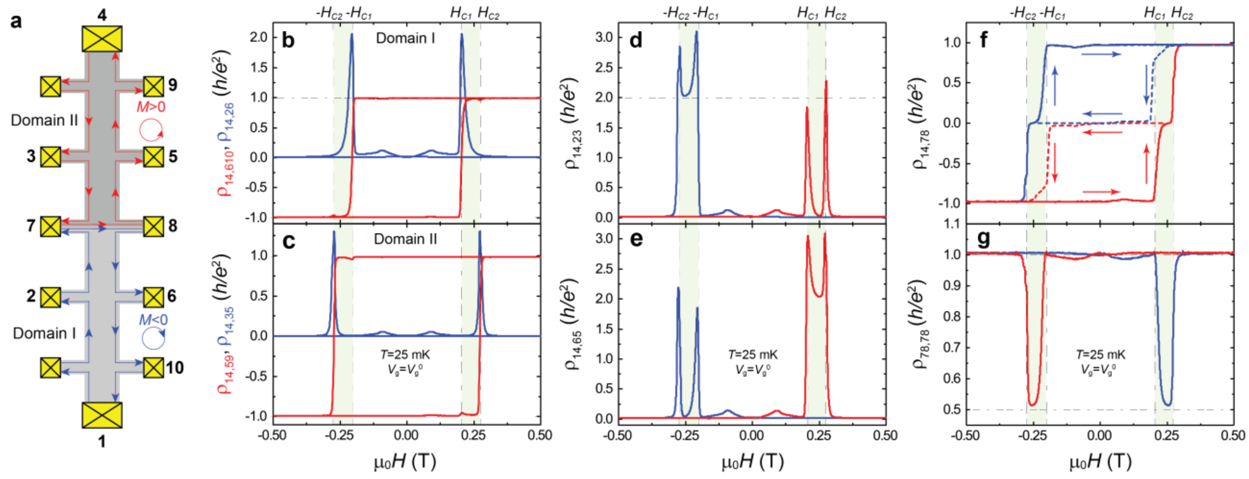

Supplementary Fig. 9| Quantized transport in the second junction between C =1 QAH and C = -1 QAH insulators. a, Schematic of chiral edge/interface channels when the current flows from contact 1 to contact 4 (i.e., from Domain I to Domain II). The red and blue lines indicate the left- and right-handed chiral edge states with upward and downward magnetization, respectively. **b, c,** $\mu_0 H$ dependence of ρ_{xx} (red) and ρ_{yx} (blue) of Domain I (**b**) and Domain II (**c**). Domain I: $\mu_0 H_{c1}$ ~0.203 T; Domain II: $\mu_0 H_{c2}$ ~0.273 T. The data in (**b**) and (**c**) are symmetrized or anti-symmetrized as a function of $\mu_0 H$ to eliminate the influence of the electrode misalignment. **d, e,** $\mu_0 H$ dependence of $\rho_{14,23}$ (**d**) and $\rho_{14,65}$ (**e**). **f,** $\mu_0 H$ dependence of the Hall resistance $\rho_{14,78}$. Unlike the measurements in Figs. 2f and 2g of the main text, contacts 7 and 8 in this device sit directly on top of the magnetic DW. The dashed lines show the minor loops of $\rho_{14,78}$. The arrows indicate the magnetic field directions in minor loop measurements. **g,** $\mu_0 H$ dependence of the two-terminal resistance $\rho_{78,78}$. All measurements are performed at $V_g = V_g^0$ and T =25 mK.

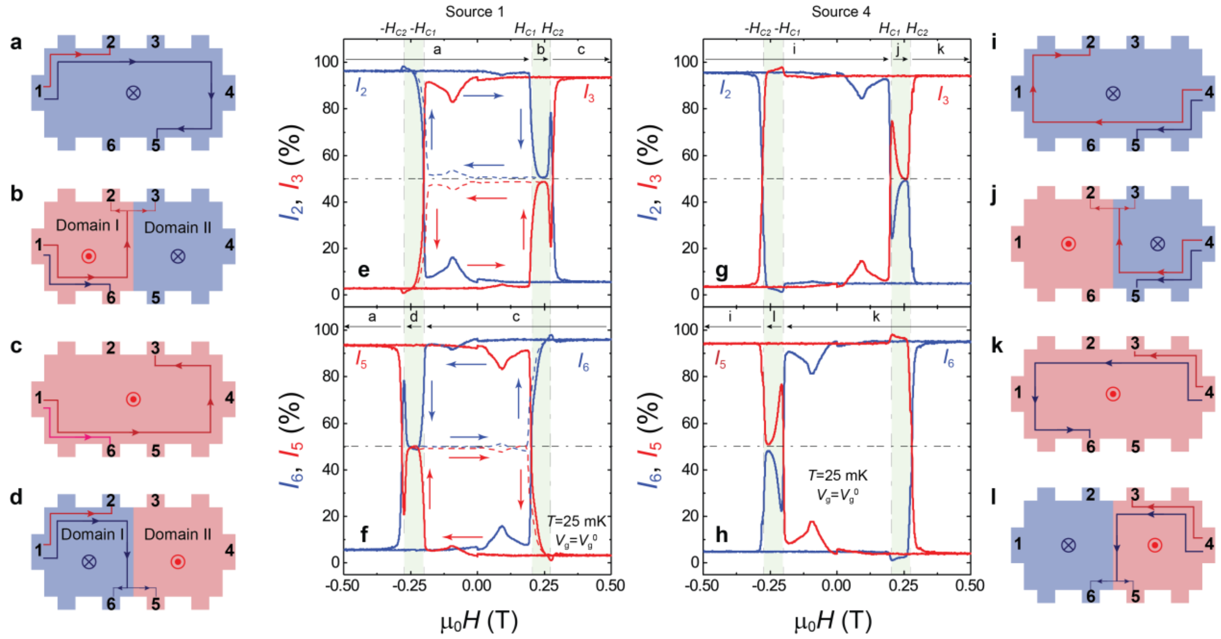

Supplementary Fig. 10| Chiral edge current distributor of the second junction between *C* =1 QAH and *C* =-1 QAH insulators. a-d, Schematics of chiral edge/interface current under different DW configurations, which are created by tuning external $\mu_0 H$. When a current of ~1 nA is injected from contact 1, the drain current measured at contact 2 or 3 with other floating contacts is shown in red, while the drain current measured at contact 5 or 6 with other floating contacts is shown in blue. **e,** $\mu_0 H$ dependence of normalized drain current for contact 2 (blue) and 3 (red). The dashed lines show the minor loops of I_2 and I_3. **f,** $\mu_0 H$ dependence of normalized drain current for contact 5 (red) and 6 (blue). The dashed lines show the minor loops of I_5 and I_6. The red and blue arrows indicate the magnetic field directions in minor loop measurements. **g, h**, Same as in (**e**) and (**f**), but for the current injected from contact 4. **i-l**, Same as in (**a**) to (**d**), but for the current injected from contact 4. All measurements are performed at $V_g = V_g^0$ and T =25 mK. The black arrows in (e to h) indicate the magnetic field sweep directions and label the DW configurations in (a to d and i to l).

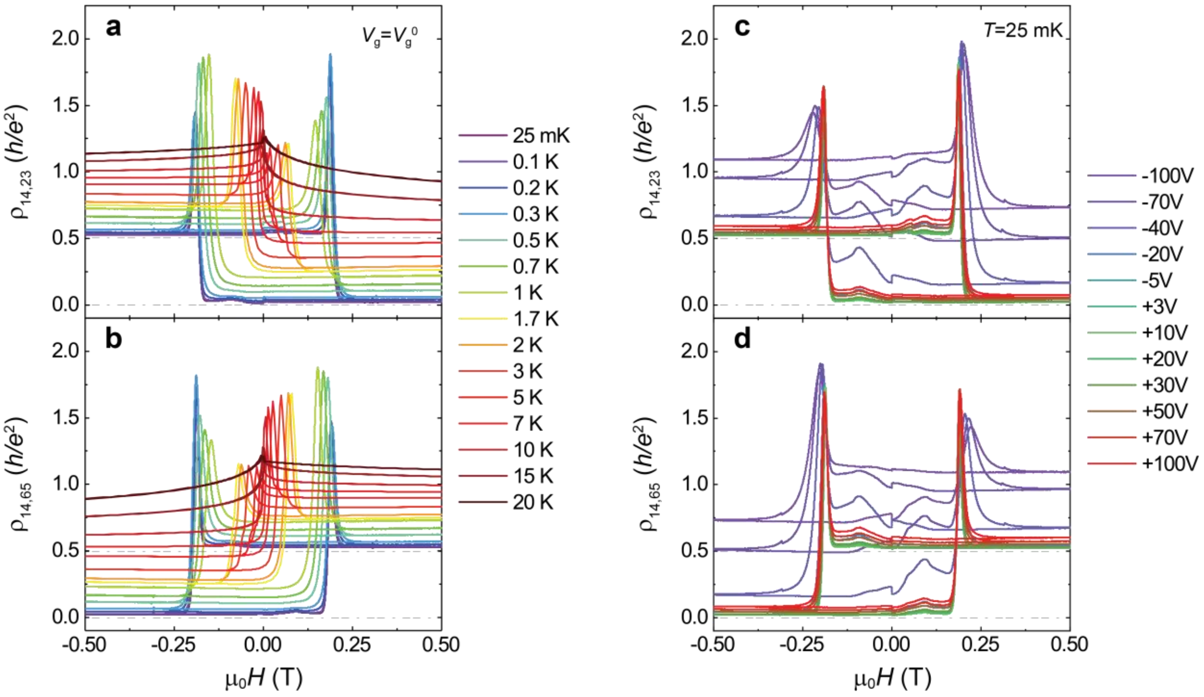

Supplementary Fig. 11| More transport results along the junction between *C* =1 QAH and *C* =2 QAH insulators. **a**, **b**, μ_0H dependence of $\rho_{14,23}$ (**a**)and $\rho_{14,65}$ (**b**) measured at different temperatures and $V_g = V_g^0$. **c-d**, μ_0H dependence of $\rho_{14,23}$ (**c**) and $\rho_{14,65}$ (**d**) measured at different gate voltages V_gs and T = 25 mK. The charge neutral point V_g^0 ~ +3 V. Schematic of the device is shown in Fig. 4b of the main text.

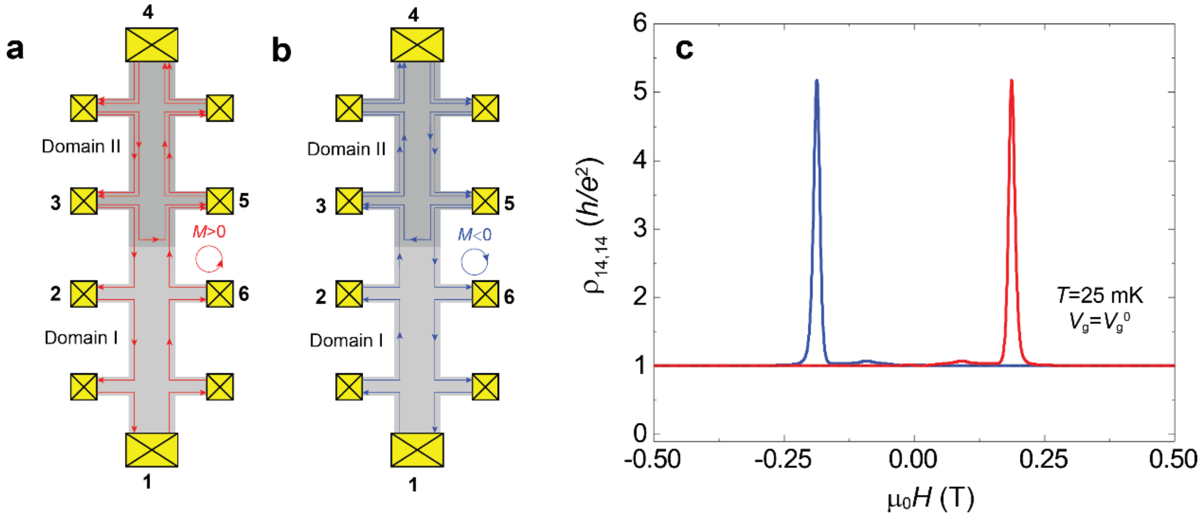

Supplementary Fig. 12| Two-terminal resistance $\rho_{14,14}$ of the junction between C =1 QAH and C =2 QAH insulators. a, b, Schematic of the chiral edge/interface channels of the junction between C =1 QAH and C = 2 QAH insulators for $M > 0$ (a) and $M < 0$ (b), respectively. **c,** μ_0H dependence of $\rho_{14,14}$ measured at T = 25 mK and $V_g = V_g^0$. The charge neutral point $V_g^0 \sim +3$ V. (a) is reused here from Fig. 4b of the main text.

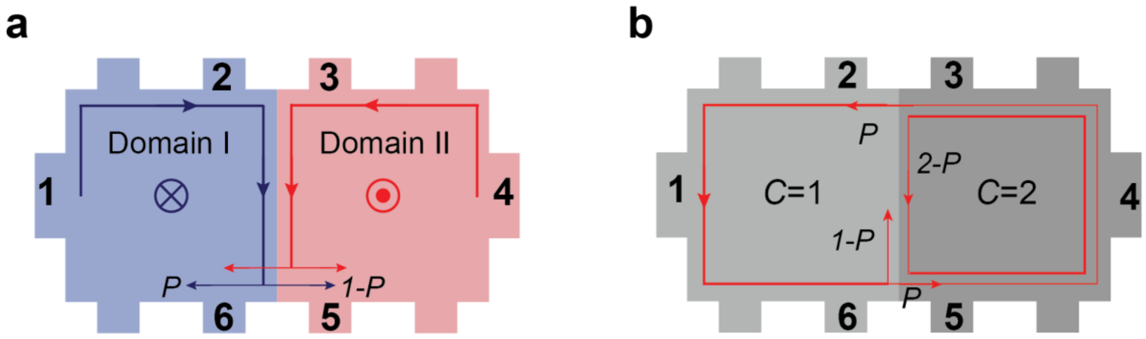

Supplementary Fig. 13| Calculating the quantized transport in QAH junctions based on the Landauer–Büttiker formalism. a, b, Schematics of chiral edge/interface current in the junction between C = 1 to C = -1 QAH insulators (**a**) and the junction between C = 1 to C = 2 QAH insulators (**b**). Here P is the CIC transmission probability through the DW.

II. Supplementary Notes

1. Quantized transport in the junction between C = 1 and C = -1 QAH insulators

To further examine the quantized transport across the magnetic DW in a C =1 QAH insulator, we measure μ_0H dependence of $\rho_{14,23}$ and $\rho_{14,65}$ at different gate voltages V_g. Supplementary Fig. 7 shows the (V_g- V_g^0) dependence of $\rho_{14,23}$ and $\rho_{14,65}$ under different DW configurations. When Domain I and Domain II have parallel magnetization alignments, as noted above, the entire sample behaves as a QAH insulator, so both $\rho_{14,23}$ and $\rho_{14,65}$ show the zero-resistance plateau near V_g=V_g^0 (Supplementary Figs. 7b and 7e). When Domain I and Domain II have antiparallel magnetization alignment, $\rho_{14,23}$ ($\rho_{14,65}$) exhibits quantized plateau at ~$2h/e^2$ and $\rho_{14,65}$ ($\rho_{14,23}$) exhibits zero resistance plateau for the Domain I-downward-Domain II-upward (Domain I-upward-Domain II-downward) state near V_g=V_g^0 (Supplementary Figs. 7a, 7c, 7d, and 7f).

In addition to the current distribution measurements when the bias current I of ~1 nA is injected at contact 1 (Fig. 4 of the main text), we also perform similar measurements on the same junction between C = 1 QAH and C = -1 QAH insulators by injecting a bias current I of ~1 nA at contact 4 (Supplementary Fig. 8). The sample shows similar behaviors, but the magnetic field where the drain currents I_2 and I_3 switch the dominance near -μ_0H_{c2} < μ_0H < -μ_0H_{c1} is different. As noted in the main text, when the bias current directly flows to one contact without passing through the magnetic DW, I_2 and I_3 (I_5 and I_6) switch the dominance instead of showing I_2 = I_3 (I_5 = I_6) when the magnetic DW appears. We find that when the bias current I is injected at contact 4, the dominance switch occurs at $\pm\mu_0H_{c2}$ (Supplementary Fig. 8). However, when the bias current I is injected at contact 1, the dominance switch occurs at $\pm\mu_0H_{c1}$ (Fig. 3 of the main text). This

implies that the drain current takes different CECs in Domain I and Domain II when the bias current I is injected at contacts 1 and 4, respectively.

Supplementary Fig. 9 shows the quantized transport results of the second junction between $C = 1$ QAH to $C = -1$ QAH insulators. We note that in this device contacts 7 and 8 sit directly on top of the magnetic DW, which is different from the device in Fig. 2 of the main text. Here, we focus on the transport data measured through contacts 7 and 8. Supplementary Fig. 9f shows the μ_0H dependence of the Hall resistance $\rho_{14,78}$. The observation of a zero Hall resistance plateau for $\mu_0H_{c1} < \mu_0H < \mu_0H_{c2}$ (and $-\mu_0H_{c2} < \mu_0H < -\mu_0H_{c1}$) validates the appearance of the two parallel CICs at the magnetic DW. By performing the minor loop measurements, we find that the zero Hall resistance plateau still appears at zero magnetic field. This validates that the two parallel CICs at the magnetic DW can persist at zero magnetic field. Supplementary Fig. 9g shows the μ_0H dependence of the two-terminal resistance $\rho_{78,78}$ along the magnetic DW. We find that $\rho_{78,78} \sim h/2e^2$ for $\mu_0H_{c1} < \mu_0H < \mu_0H_{c2}$, further confirming that two parallel CICs are created near the magnetic DW.

2. Nonuniform thickness influence on CICs

For the junction between $C = +1$ and $C = -1$ QAH insulators (Fig. 1b), since both QAH insulators have one CES, the additional 2 QL $(Bi, Sb)_{1.78}V_{0.22}Te_3$ layer in Domain II is unlikely to influence the two CICs near the magnetic DW (Figs. 1a and 2a). The observation of the ~50% transmission probability of the CIC confirms that the two CICs near the magnetic DW are equivalent (Fig. 2f). For the junction between $C = 1$ and $C = 2$ QAH insulators, the additional 3 QL $(Bi, Sb)_{1.74}Cr_{0.26}Te_3$/4 QL $(Bi, Sb)_2Te_3$ layer in Domain II (i.e., the $C = 2$ QAH insulator) creates the second CES, which propagates along the DW between $C = 1$ and $C = 2$ QAH insulators.

Therefore, the nonuniform thickness of the QAH junction is also unlikely to influence the single CIC near the DW between $C = 1$ and $C = 2$ QAH insulators (Fig.4b).

3. Quantized transport behavior in electron-doped regime

For magnetically doped TI thin films, the charge neutral point (i.e. V_g=V_g^0) is close to the bulk valence band maximum, but far away from the bulk conduction band minimum. This asymmetric band structure results in the different gate-dependent transport behaviors between V_g>V_g^0 and V_g< V_g^0 (Refs. [1-4]). For V_g< V_g^0, the chemical potential first crosses the bulk valence bands, and thus plenty of carriers are introduced, which leads to a large deviation from the QAH state. However, for V_g> V_g^0, the chemical potential first crosses the helical surface states, which introduces far fewer carriers and thus does not affect the QAH state as much. This is the reason that the quantized transport persists in a large range of gate voltage for V_g>V_g^0 (Supplementary Fig. 7).

4. Calculating the quantized transport in QAH junctions based on the Landauer–Büttiker formalism

Based on the Landauer–Büttiker formalism[2,5], we separately calculate the quantized transport in the two QAH junctions in the main text (i.e. the junction between $C = 1$ QAH and $C = -1$ QAH insulators and the junction between $C = 1$ QAH and $C = 2$ QAH insulators).

(1) The junction between $C = 1$ and $C = -1$ QAH insulators:

For the Domain I-downward-Domain II-upward state (Supplementary Fig. 13a), the two parallel CICs propagate at the magnetic DW. We define the CIC transmission probability through the magnetic DW as P. We can write the Landauer–Büttiker formalism for this QAH junction as:

$$\begin{pmatrix} I_1 \\ I_2 \\ I_3 \\ I_4 \\ I_5 \\ I_6 \end{pmatrix} = \begin{pmatrix} G_0 & 0 & 0 & 0 & 0 & -G_0 \\ -G_0 & G_0 & 0 & 0 & 0 & 0 \\ 0 & 0 & G_0 & -G_0 & 0 & 0 \\ 0 & 0 & 0 & G_0 & -G_0 & 0 \\ 0 & -PG_0 & -(1-P)G_0 & 0 & G_0 & 0 \\ 0 & -(1-P)G_0 & -PG_0 & 0 & 0 & G_0 \end{pmatrix} \begin{pmatrix} V_1 \\ V_2 \\ V_3 \\ V_4 \\ V_5 \\ V_6 \end{pmatrix}$$

By solving these equations, we achieve the corresponding quantized resistances of the junction between *C* = 1 QAH and *C* = -1 QAH insulators:

$$\rho_{14,62} = -\frac{h}{e^2}$$

$$\rho_{14,53} = \frac{h}{e^2}$$

$$\rho_{14,23} = \frac{1}{P}\frac{h}{e^2}$$

$$\rho_{14,65} = \frac{1-2P}{P}\frac{h}{e^2}$$

$$\rho_{14,14} = \frac{1}{P}\frac{h}{e^2}$$

For the CIC transmission probability *P*~0.5, $\rho_{14,23}$, $\rho_{14,65}$, and $\rho_{14,14}$ are found to be ~$2h/e^2$, ~0, and ~$2h/e^2$, respectively. All these values are in good agreement with our experimental results (Fig. 2 and Supplementary Figs. 6, 7, 9). Our results are also consistent with two fully mixed CICs in quantum Hall systems [6-8].

(2) The junction between *C* = 1 to *C* = 2 QAH insulators:

Next, we calculate the quantized transport in the junction between *C* = 1 to *C* = 2 QAH insulators. As noted above, we define the CIC transmission probability through the magnetic DW as *P*. For *M*>0, we can write the Landauer–Büttiker formalism for this QAH junction as:

$$\begin{pmatrix} I_1 \\ I_2 \\ I_3 \\ I_4 \\ I_5 \\ I_6 \end{pmatrix} = \begin{pmatrix} G_0 & -G_0 & 0 & 0 & 0 & 0 \\ 0 & G_0 & -PG_0 & 0 & 0 & -(1-P)G_0 \\ 0 & 0 & 2G_0 & -2G_0 & 0 & 0 \\ 0 & 0 & 0 & 2G_0 & -2G_0 & 0 \\ 0 & 0 & -(2-P)G_0 & 0 & 2G_0 & -PG_0 \\ -G_0 & 0 & 0 & 0 & 0 & G_0 \end{pmatrix} \begin{pmatrix} V_1 \\ V_2 \\ V_3 \\ V_4 \\ V_5 \\ V_6 \end{pmatrix}$$

By solving these equations, we achieve the corresponding quantized resistances of the junction between $C = 1$ QAH and $C = 2$ QAH insulators:

$$\rho_{14,62} = \frac{h}{e^2}$$

$$\rho_{14,53} = \frac{h}{2e^2}$$

$$\rho_{14,23} = \frac{1-P}{P}\frac{h}{e^2}$$

$$\rho_{14,65} = \frac{2-P}{2P}\frac{h}{e^2}$$

$$\rho_{14,14} = \frac{1}{P}\frac{h}{e^2}$$

We note that the $C = 1$ and $C = 2$ QAH insulators in our device have the same $\mu_0 H_c$ and always have the parallel magnetization alignment, so the two QAH insulators will share the same CES chirality. Therefore, for the CIC transmission probability P~1, $\rho_{14,23}$, $\rho_{14,65}$, and $\rho_{14,14}$ are found to be ~0, ~ $h/2e^2$, and ~ h/e^2, respectively. All these values are in good agreement with our experimental results (Fig. 4 and Supplementary Figs. 11 and 12).

Supplementary References

1 Chang, C.-Z., Liu, C.-X. & MacDonald, A. H. Colloquium: Quantum anomalous Hall effect. *arXiv:2202.13902* (2022).

2 Chang, C. Z., Zhao, W. W., Kim, D. Y., Wei, P., Jain, J. K., Liu, C. X., Chan, M. H. W. & Moodera, J. S. Zero-Field Dissipationless Chiral Edge Transport and the Nature of Dissipation in the Quantum Anomalous Hall State. *Phys. Rev. Lett.* **115**, 057206 (2015).

3 Li, W., Claassen, M., Chang, C. Z., Moritz, B., Jia, T., Zhang, C., Rebec, S., Lee, J. J., Hashimoto, M., Lu, D. H., Moore, R. G., Moodera, J. S., Devereaux, T. P. & Shen, Z. X. Origin of the Low Critical Observing Temperature of the Quantum Anomalous Hall Effect in V-Doped (Bi, $Sb)_2Te_3$ Film. *Sci. Rep.* **6**, 32732 (2016).

4 Wang, W. B., Ou, Y. B., Liu, C., Wang, Y. Y., He, K., Xue, Q. K. & Wu, W. D. Direct Evidence of Ferromagnetism in a Quantum Anomalous Hall System. *Nat. Phys.* **14**, 791-795 (2018).

5 Landauer, R. Spatial Variation of Currents and Fields Due to Localized Scatterers in Metallic Conduction. *IBM J. Res. Dev.* **1**, 223-231 (1957).

6 Williams, J. R., DiCarlo, L. & Marcus, C. M. Quantum hall effect in a gate-controlled p-n junction of graphene. *Science* **317**, 638-641 (2007).

7 Abanin, D. A. & Levitov, L. S. Quantized transport in graphene p-n junctions in a magnetic field. *Science* **317**, 641-643 (2007).

8 Ozyilmaz, B., Jarillo-Herrero, P., Efetov, D., Abanin, D. A., Levitov, L. S. & Kim, P. Electronic transport and quantum hall effect in bipolar graphene p-n-p junctions. *Phys. Rev. Lett.* **99**, 166804 (2007).
